# British Home for Incurables, Clapham

**Published:** 1893-11-25

**Authors:** 


### HOSPITAL MEETINGS.

### BRITISH HOME FOR INCURABLES, CLAPHAM.

The half-yearly meeting of the governors and subscribers to this institution was held on Thursday last at the City Terminus Hotel, Cannon Street, when the chair was taken by Mr. F. A. Bevan, in the absence of the President, Lord Amhurst. It was announced that the new home at Streatham was being quickly completed, and would be opened, it was hoped, in July next. The Chairman appealed for funds to provide for the furnishing of the new home, for which a sum of £2,000 would be needed. A generous donation had been received of £1,500 to provide for the building of a new chapel.

